# Incubation of ovine scrapie with environmental matrix results in biological and biochemical changes of PrP^Sc^ over time

**DOI:** 10.1186/s13567-015-0179-y

**Published:** 2015-05-01

**Authors:** Ben C Maddison, John Spiropoulos, Christopher M Vickery, Richard Lockey, Jonathan P Owen, Keith Bishop, Claire A Baker, Kevin C Gough

**Affiliations:** ADAS UK, School of Veterinary Medicine and Science, The University of Nottingham, Sutton Bonington Campus, College Road, Sutton Bonington, Leicestershire, UK; Animal and Plant Health Agency, Woodham Lane, New Haw, Addlestone, Surrey, UK; Current address: University of Southampton, Southampton, SO17 1BJ UK; School of Veterinary Medicine and Science, The University of Nottingham, Sutton Bonington Campus, College Road, Sutton Bonington, Leicestershire, UK

## Abstract

Ovine scrapie can be transmitted via environmental reservoirs. A pool of ovine scrapie isolates were incubated on soil for one day or thirteen months and eluted prion was used to challenge tg338 mice transgenic for ovine PrP. After one-day incubation on soil, two PrP^Sc^ phenotypes were present: G_338_ or Apl_338_ii. Thirteen months later some divergent PrP^Sc^ phenotypes were seen: a mixture of Apl_338_ii with either G_338_ or P_338,_ and a completely novel PrP^Sc^ deposition, designated Cag_338_. The data show that prolonged ageing of scrapie prions within an environmental matrix may result in changes in the dominant PrP^Sc^ biological/biochemical properties.

## Introduction, methods and results

Prion diseases (or transmissible spongiform encephalopathies, TSEs) are fatal, progressive neurological disorders that have no effective treatment or cure. Prion diseases include human Creutzfeldt-Jakob disease (CJD), bovine spongiform encephalopathy (BSE), scrapie in sheep and goats, and chronic wasting disease (CWD) in deer and elk. The prion hypothesis states that the causal agent is a misfolded version of the cellular prion protein (PrP^C^), termed PrP^Sc^ [1].

It is known that particular prion diseases can include strains that display distinct and reproducible disease phenotypes. It is most likely that the prion agent is not a single entity but is made up of a plethora of different conformers of PrP^Sc^ and the dominant PrP^Sc^ conformation causes the specific disease characteristics for a particular infection including pathology, clinical signs and PrP^Sc^ molecular signatures [2]. The identification of prion strains is in fact therefore a description of the dominant disease characteristics. The “gold standard” method employed to define scrapie strains is mouse bioassay using either wild type or preferably transgenic mice such as the tg338 line [3]. These transgenic mice overexpress an ovine PrP transgene and display high sensitivity and specificity to ovine scrapie prions. Of the several phenotypic parameters exhibited in a host species which are used to discriminate TSEs, PrP^Sc^ distribution in the brain detected by immunohistochemistry (IHC) or PET/Histo-blot offers the highest discriminatory power and it can be applied on an individual mouse basis [4-8]. This biological property of prions in conjunction with analysis of the biochemical properties of the agent recovered from the same host species offer a powerful means to identify TSE strains even when they are applied at primary passage as they remain essentially unchanged through serial passages [4,6,9,10].

Scrapie is effectively transmitted between susceptible sheep and goats by animal-to-animal contact and via environmental reservoirs, a disease trait that is shared with CWD in deer/elk. For both diseases, the agent is disseminated widely in vivo and excreted/secreted via multiple routes (reviewed in [11]). The likely location of environmental reservoirs are water, soil, metal surfaces, wood surfaces and concrete surfaces ([12,13] reviewed in [14]). Furthermore, environmental prion is stable and remains infectious for years [15].

The purpose of the present study was to assess the viability of scrapie prions in a soil matrix over time. Pools of hindbrain from nine scrapie-infected sheep with VRQ/VRQ (amino acid positions 136, 156 and 171 respectively) *PRNP* genotypes and twenty genotype matched scrapie-free controls were made into homogenates and applied to soil columns containing a sandy loam soil as previously described [16]. Soil columns were kept at 16–20 °C and constant water content and sampled 1 day and 13 months after the addition of the prion sample. Equivalent samples were taken for soil incubated with the prion-free control sample, and soil unexposed to brain material was used as a further control. All soil was removed from a column and homogenised by mixing. Prion protein was then extracted from soil [15]: 100 mg of soil was re-suspended for 1 h in 500 μL PBS prior to centrifugation at 800 *g* for 10 min. The soil pellet was re-suspended in 100 μL of 1% (w/v) SDS in PBS and shaken vigorously for 1 h. After centrifugation at 800 *g* for 10 min prion protein in the supernatant was removed. Extracted prion was then precipitated for 30 min at 37 °C by the addition of 15 μL of 4% (w/v) sodium phosphotungstic acid (Napta) and 170 mM magnesium chloride, and recovered by centrifugation at 12 100 *g* for 30 min. The pellet was air-dried and re-suspended in 20 μL sterile saline. Each inoculum (20 μL) was used to challenge intracranially 5–10 week old tg338 mice (*n* = 10) as described previously [9]. After inoculation the mice were monitored for signs of neurological disease and were euthanized after exhibiting clinical signs. All animal work was approved by the Animal and Plant Health Agency local ethics committee and was carried out in accordance with the Animals (Scientific Procedures) Act 1986 under Home Office project license 70/6310. In combination with clinical signs, immunohistochemical (IHC) analysis was used to diagnose prion infection for all mice in the study. After euthanasia the brain of each mouse was removed and processed as described previously [9]. TSE diagnosis was based on PrP^Sc^ detection in brain sections with polyclonal antibody Rb486 following a standard protocol [17]. Identification of different PrP^Sc^ types and their distribution in the murine brain was used to identify defined PrP^Sc^ distribution patterns as described previously for wild type and transgenic mice [4-7]. Slides were analysed blind by two independent observers (J.S. and C.V.); the agreement between the two observers regarding PrP^Sc^ deposition pattern identification was 100%.

**Figure 1:**
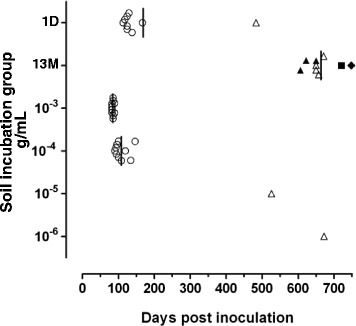
**Incubation periods of mice after challenge with scrapie.** Mice were inoculated with extracts of soil incubated with a pool of classical scrapie isolates or with a dilution series of the same scrapie pool without soil incubation g/mL of brain inoculum are shown). Open circles: the PrP^Sc^ distribution phenotype G_338_; open triangles: Apl_338_ii; square: Cag_338_; solid triangles: G_338_/Apl_338_ii mix; diamond: P_338_/Apl_338_ii mix. Each vertical line indicates the mean incubation period of a group of mice irrespective of their PrP^Sc^ distribution phenotype. 1D and 13 M are the prion eluted from soil after a 1 day or 13 month incubation respectively.

**Figure 2:**
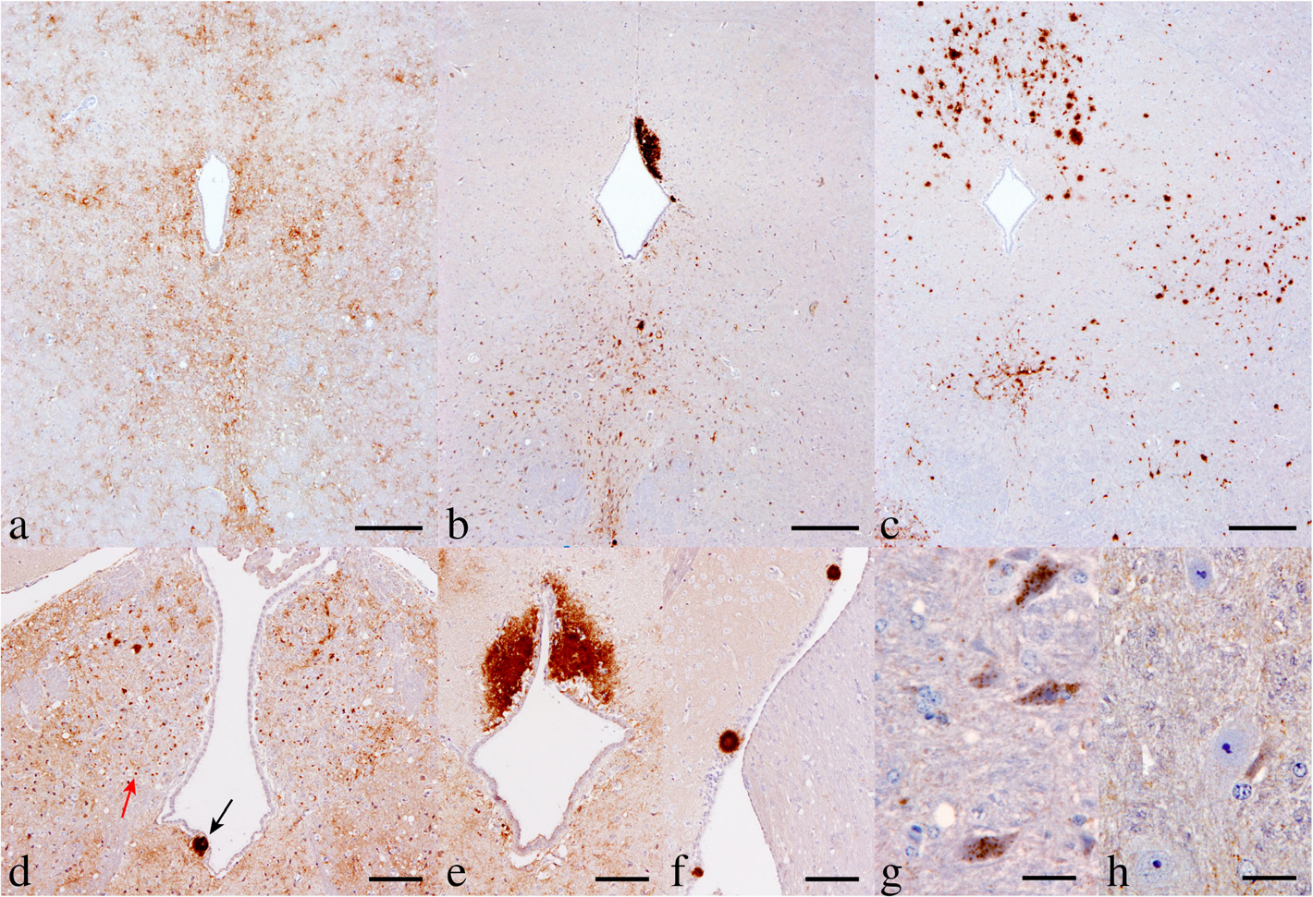
**Representative images of PrP** ^**Sc**^ **deposition in the midbrain of tg338 mice after challenge with scrapie.** The deposition patterns observed were characteristic of strains G_338_ **(a)**, Apl_338_ii **(b)** and Cag_338_ **(c)**. Three mice showed phenotypic characteristics typical of both G_338_ and Apl_338_ii patterns; photos d-f are all from a single mouse showing small discrete aggregates (**d**, red arrow) in the habenular bodies which is a G_338_ characteristic feature in addition to plaques and large aggregates in subpial areas (**d**, black arrow; **e**, periaqueductal grey matter; **f**, lateral ventricle) which are associated with Apl_338_ii. In a single mouse with Apl_338_ii phenotype, punctate and prominent intraneuronal labelling **(g)** which are features associated with the P_338_ pattern were also observed; the Apl_338_ii phenotype is not usually associated with intraneuronal labelling **(h)**. The neurons in **g** and **h** are located in the olive nucleus. Scale bar in **a**-**c** represents 250 μm; **d**–**f** 100 μm; **g** and **h** 25 μm.

With inoculum extracted from soil incubated with the scrapie sample for 1 day, eight out of the 10 challenged mice succumbed to TSE with incubation periods <170 days post inoculation (dpi) (Figure 1A). IHC analysis of the mice revealed granular PrP^Sc^ deposits distributed mainly along the brainstem, thalamus and basal ganglia with little involvement of the cerebral or cerebellar cortex; this pattern has been previously designated as G_338_ (Figure 2A) [4]. One further mouse died 483 dpi (Figure 1A) and the main PrP^Sc^ pattern feature was plaques and large aggregates of PrP^Sc^ in the brain parenchyma and perivascular plaques in round meningeal vessels (Figure 2B), a distribution pattern previously recorded as Apl_338_ii. One mouse that died 243 dpi was TSE negative and was treated as an intercurrent death. No mice displayed signs of TSE between 170–483 dpi.

After 13 months incubation of the scrapie sample on soil, prions were extracted and inoculated into tg338 mice. One mouse was diagnosed TSE negative (intercurrent death) and the remaining nine were TSE positive. The incubation period of the TSE positive mice was 606–748 dpi (Figure 1A). With one exception all mice showed a pattern that was compatible with Apl_338_ii. This was the only pattern observed in four mice, while in another three it was observed in conjunction with G_338_ (Figures 2D-F) and in a single mouse signs of Apl_338_ii and P_338_ were observed concomitantly (Figure 2G). P_338_ is a pattern characterised by punctate deposits in the neuropil and prominent well-defined intraneuronal PrP^Sc^ accumulations as described previously [4]. One mouse also showed a previously unrecognised PrP^Sc^ pattern designated Cag_338_ characterised by granular PrP^Sc^ deposits, which increased in intensity multifocally, to give rise to coalescing aggregates in the neuropil (Figure 2C). The areas affected more extensively were the midbrain and the medulla.

**Figure 3:**
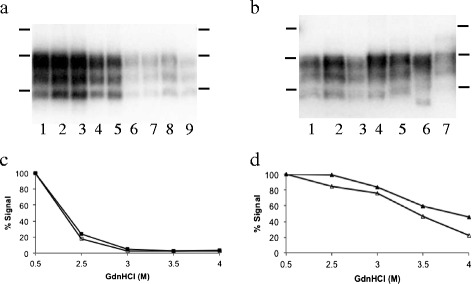
**PrP** ^**Sc**^ **characteristics of the scrapie isolates.** Nine ovine hindbrain samples that were pooled and incubated with soil **(a)** were analysed by western blot. Murine isolates **(b)** designated G_338_ (lanes 1, 2 and 3), Apl_338_ii (lane 4), Apl_338_ii/G_338_ mixed phenotype (lane 5), Apl_338_ii/P_338_ mixed phenotype (Lane 6) and Cag_338_ (Lane 7) are also shown. Blots were probed with anti-PrP antibody SHa31 and molecular mass markers of 20, 30 and 40 kDa are indicated. All samples were analysed twice to determine the molecular mass of unglycosylated PrP^Sc^ and gave consistent results. The strains G_338_ **(c)** and Apl_338_ii **(d)** were further analysed by the conformational stability assay and gave distinct profiles. These molecular traits were consistent both before (closed symbols) and after (open symbols) treatment with SDS and Napta. Analysis was carried out on 3 murine isolates of G_338_ and 2 murine isolates of Apl_338_ii and the presented data is representative of these isolates.

Previous studies have shown that the P_338_ IHC presentation of PrP^Sc^ is accompanied by a relatively low molecular weight for the PK-resistant PrP^Sc^ compared to both Apl_338_ii and G_338_ [4,6]. We looked to investigate whether the distinct IHC presentations described here were accompanied by distinct PrP^Sc^ properties. Both the original sheep samples and the murine samples were digested with PK, analyzed on western blots and the prion detected with the antibody SHa31 as previously described [18] (Figures 3A and B). The results show that all ovine samples had indistinguishable PrP^Sc^ profiles with an unglycosylated PrP^Sc^ size of 19.0 +/− 0.3 kDa. The G_338_, Apl_338_ii and G_338_/Apl_338_ii mixed IHC phenotypes had a similar size of 19.1 +/− 0.3 kDa. However, the Apl_338_ii/P_338_ mixed phenotype had a relatively low molecular mass by comparison of 17.1 kDa, consistent with previously published data for P_338_ [4,6]. Also, the unglycosylated PrP^Sc^ of Cag_338_ had a relatively high molecular mass of 20.6 kDa (Figure 3B). In addition, we determined that the G_338_ and Apl_338_ii IHC phenotypes could be readily distinguished by the stability of their PrP^Sc^ (Figures 3C and D). The assay was carried out as described previously [19]. Briefly, aliquots of each murine brain homogenate were incubated with increasing molar concentrations of GdnHCl (final concentrations of 0.5, 2.5, 3.0, 3.5, and 4 M) 1 h at 37 °C. Subsequently all samples were adjusted to a final GdnHCl concentration of 0.4 M, proteinase K was added to a final concentration of 50 μg/mL and the samples incubated for 1 h at 37 °C. Reactions were stopped with 5 mM PMSF. Samples were analysed by western blot using antibody SHa31. The level of signal for each PrP triplet treated with 2.5, 3.0, 3.5 or 4 M GdnHCl was expressed as a percentage of the signal for the same sample treated with 0.5 M. G_338_ was more susceptible to GdnHCl treatment becoming PK sensitive after treatment with 3 M of the denaturant. In contrast, Apl_338_ii was relatively stable to denaturation with readily detectable PK-resistant PrP^Sc^ after treatment with 4 M GdnHCl (Figures 3C and D). Overall, the PrP^Sc^ biochemical characteristics were distinct for each of Apl_338_ii, G_338_, Apl_338_ii/P_338_ mixed and Cag_338_ IHC phenotypes.

Extracts from soil unexposed to brain material and soil treated with scrapie-free brain homogenate were bioassayed in tg338 mice and were TSE negative.

For comparison, the original scrapie sample without any incubation with soil was also titrated in tg338 mice over a range of 10-fold dilutions (*n* = 10 mice for each dilution; Figure 1B). All mice challenged with 20 μg and 2 μg scrapie brain succumbed to scrapie with a G_338_ IHC phenotype. Only one of the 10 mice challenged with either 200 or 20 ng of brain pool was diagnosed with scrapie and in each case this produced an Apl_338_ii IHC phenotype. Challenge with lower amounts of brain pool did not cause disease. Prion desorbed from soil after 1 day displayed the same IHC and PrP^Sc^ phenotypes as the original scrapie pool, that is Apl_338_ii and G_338_. However, it is possible that the extraction and precipitation treatments have an effect on PrP^Sc^ phenotype or recovery. To test this, murine brain homogenates for G_338_ and Apl_338_ii phenotypes (10% w/v; 100 μL) were diluted to 200 μL with 2% (w/v) SDS and shaken vigorously for 1 h. After centrifugation at 800 *g* for 10 min prion in the supernatant was precipitated with Napta and brain homogenate and Napta precipitate were analyzed by western blot as detailed above and the total signal for the PrP triplet was determined by densitometry. The percentage recovery after SDS treatment/Napta precipitation for each isolate was determined and comparison of the recoveries of G_338_ and Apl_338_ii was carried out using a two-tailed students *t*-test. The percentage recoveries for 3 isolates of murine G_338_ and 2 isolates of murine Apl_338_ii were determined and the mean recoveries were 57 and 56% respectively, differences in the recoveries of the two PrP^Sc^ phenotypes were not significant (*p* = 0.97). In addition, the molecular phenotypes were maintained before and after SDS/Napta treatment (Figures 3C and D).

## Discussion

The bioassay data show that the hit rate was equivalent for sheep scrapie extracted from soil after 1 day or 13 months incubation indicating ovine scrapie infection was retained on soil over a prolonged time period. The data also clearly suggest that between day 1 and month 13 the biological and biochemical properties of the prion that is desorbed from soil change considerably. In our view, this concomitant change of biological and biochemical properties as described here is indicative of strain variation. Indeed many of these phenotypic characteristics have been attributed to characterised strains isolated and studied in tg338 mice (Table 1) [4,6]. Some of the strain phenotypes identified after a 13 month incubation are highly novel. Both G_338_ and P_338_ strains are relatively fast incubation strains and have not been reported before in a mixed phenotype with Apl_338_ii or at these prolonged incubation times. The identification particularly of G_338_ IHC characteristics, in conjunction with Apl_338_ii, in the brains of mice showing incubation period >600 dpi is intriguing as the maximum incubation period associated with G_338_ is known to be <200 dpi [20]. Therefore the possibility that G_338_ was existing as an independent entity in the inoculum used to challenge the mice is unlikely even if we accept that in the presence of a significantly slower strain, such as Apl_338_ii, the propagation of G_338_ was delayed. Another possible explanation would be that agents with G_338_ or P_338_ properties could emerge from Apl_338_ii at a later stage of the incubation period. Alternatively the G_338_ and P_338_ phenotypic characteristics that were observed in conjunction with Apl_338_ii indicate phenotypes that have some G_338_ or P_338_ properties associated with unusually prolonged incubation periods. Without isolating each of these agents in a pure state to study their properties it is not possible to draw definitive conclusions regarding their exact strain characteristics. However, their existence at this stage, particularly of the P_338_ IHC phenotype, which is also accompanied with biochemical properties that are attributed to the P_338_ strain, cannot be ignored and adds valuable information regarding the diversity of scrapie phenotypes that can emerge after prolonged incubation period with soil. The Cag_338_ strain phenotype is reported here for the first time. Collectively, therefore, these data suggest that the ageing of prions within an environmental matrix can affect their biological and biochemical properties suggesting strain alterations. The three novel phenotypes of desorbed prion strains observed after 13 months incubation on soil were not detected in a range of 10-fold dilutions of the original scrapie sample and the SDS/Napta treatment of samples to desorb them from soil had no apparent effect on G_338_ or Apl_338_ii recovery or PrP^Sc^ phenotype. Therefore, these novel PrP^Sc^ presentations must be a consequence of their interaction with soil or ageing or both. It is not known whether this emergence of novel phenotypes seen here during ageing on soil reflects the selection of existing conformers present in the original sample or *de novo* mutation to produce novel conformations of the prion. The study compared PrP^Sc^ phenotypes that are recovered from soil after 1 day and 13 month periods and the effects of soil interaction and incubation time alone are not considered separately. Therefore it is also not known whether the observed changes in dominant prion strains are dictated by incubation time at ambient temperature alone or by interaction with soil over a prolonged period. However, regardless of the mechanisms of the observed ageing, the unequivocal finding is that when a mixture of prion phenotypes are added to a soil environment the dominant pathologies change over time. Whether analogous ageing of prions occurs in other natural environments that may harbour prion reservoirs remains to be established. The presence of “dynamic” reservoirs of environmental scrapie infectivity could possibly lead to the emergence of novel strains of scrapie in natural infections. Such events may have contributed to the significant (and unusual) diversity of the scrapie disease agent.

**Table 1:** **Main features of characterised strains identified in this study** [4,6]

		**Main PrP** ^**Sc**^ **characteristics**	
**Strain**	Incubation period (IP)*	**Immunohistochemistry (IHC)**	**Western blot** ^♯^
G_338_	78 ± 6	Granular deposits mainly in brain stem and thalamus	21 KDa
P_338_	153 ± 8	Punctate and intraneuronal deposits mainly in brain stem and thalamus	19 KDa
Apl_338_ii	249 ± 59	Aggregates and plaques. Midbrain is most affected area	21 KDa

*Incubation period (IP) values denote mean ± SD and indicate days post inoculation (dpi) after three serial passages. At first passage IP values of certain strains such as Apl_338_ may be prolonged [6]. G_338_ is an exceptional strain as shorter IP values can be observed at primary passage [21] with serial passages resulting in IP prolongation by approximately 12 dpi.

^♯^Western blot values refer to the molecular mass of the unglycosylated band. Absolute values may differ depending on the western blot conditions. However, the P_338_ unglycosylated band always migrates further indicating a 2 KDa lower molecular mass compared to G_338_ and Apl_338_ii.

## Abbreviations

TSE: Transmissible spongiform encephalopathy
CWD: Chronic wasting disease
PrP/PrP^C^: Cellular prion protein
PrP^Sc^: Disease-associated prion protein
IHC: Immunohistochemistry
Napta: Sodium phosphotungstic acid
